# Characterizing pre-dialysis care in the era of eGFR reporting: a cohort study

**DOI:** 10.1186/1471-2369-12-12

**Published:** 2011-03-15

**Authors:** Khaled Abdel-Kader, Gary S Fischer, James R Johnston, Chen Gu, Charity G Moore, Mark L Unruh

**Affiliations:** 1 Renal-Electrolyte Division, University of Pittsburgh, Pittsburgh, USA; 2 Department of General Internal Medicine, University of Pittsburgh, Pittsburgh, USA

## Abstract

**Background:**

Chronic kidney disease (CKD) is a common disorder associated with increased morbidity and mortality. Primary care physicians (PCPs) care for the majority of pre-dialysis CKD patients; however, PCPs often do not recognize the presence of CKD based on serum creatinine levels. Prior studies suggest that PCPs and nephrologists deliver suboptimal CKD care. One strategy to improve disease awareness and treatment is estimated glomerular filtration rate (eGFR) reporting. We examined PCP and nephrologist CKD practices before and after routine eGFR reporting.

**Methods:**

We conducted a retrospective cohort study of patients with CKD 3b-4 (eGFR < 45) seen at a university-based, outpatient primary care clinic. Using a chi-square or Fisher's exact test, we compared co-management rates, renal protective strategies, CKD documentation, and laboratory processes of care in 274 patients and 266 patients seen in a 6-month period prior to and following eGFR implementation, respectively.

**Results:**

CKD co-management increased from 22.6% pre-eGFR to 48.5% post-eGFR (P < 0.0001). eGFR reporting did not improve angiotensin converting enzyme inhibitor or angiotensin receptor blocker use or quantitative urinary testing. However, non-steroidal anti-inflammatory drug avoidance (pre-eGFR 81.8% vs. post- eGFR 90.6%, P = 0.003) and phosphorus and parathyroid hormone testing improved (pre-eGFR vs. post-eGFR: 32.5% vs. 51.5%, P < 0.0001; 12.4% vs. 36.1%, P < 0.0001 respectively).

**Conclusions:**

A marked increase in CKD co-management was observed following eGFR implementation. Although some improvements in processes of care were noted, this did not include angiotensin converting enzyme inhibitor or angiotensin receptor blocker use. Overall care remained suboptimal despite eGFR reporting; further strategies are needed to improve PCP and nephrologist CKD care.

## Background

Over 12 million adults in the United States (US) have chronic kidney disease (CKD) stage 3 or greater (estimated glomerular filtration rate < 60 ml/min/1.73 m^2^) [1]. Further, the prevalence of CKD appears to be rising [1] and with the increasing incidence of obesity, diabetes, and hypertension [2-6], this trend is expected to continue. Presently, primary care physicians (PCPs) deliver most of the care to patients with non-dialysis dependent CKD [7-10]. However, PCPs may not recognize the presence of CKD and as many as 66% of PCPs may be unaware of Kidney Disease Outcome Quality Initiative (K/DOQI) clinical practice guidelines [11]. Given that CKD progression and its associated morbidity and mortality can be reduced with optimal care [12,13], late detection and treatment contributes to poor patient outcomes [14]. Among the approaches advocated for the improvement of CKD outcomes are optimization of care by increasing disease awareness and adherence to CKD clinical practice guidelines including angiotensin converting enzyme inhibitor (ACEI) or angiotensin receptor blocker (ARB) use and timely renal referrals.

One important development in the ongoing effort to improve CKD recognition is the implementation of routine eGFR reporting. Although imperfect, eGFR values account for demographic factors that are important determinants of muscle mass and serum creatinine levels and provide physicians with more accurate estimates of kidney function than isolated serum creatinines [15]. The National Kidney Foundation has advocated for the use of prediction equations to estimate kidney function [16] and the most widely used equation is presently the 4-variable Modification of Diet in Renal Disease (MDRD) Study equation [17-21]. The routine reporting of eGFR values with serum creatinine has been advocated by some experts as an important approach to improve CKD awareness and treatment [22-24] and recent data reveal that an increasing number of US labs are adopting universal eGFR reporting [21,25]. Multiple studies have documented the effect of routine eGFR reporting on renal referrals [8,26-28]; however, few studies have examined the quality of care delivered to CKD patients since the implementation of eGFR reporting [28-30]. In addition, despite previously well-documented deficiencies in nephrologist care of CKD patients [10,31-35], few studies have examined whether there have been improvements in the care of co-managed CKD patients following eGFR reporting.

In this report, we examine the care of patients with advanced CKD (stages 3b-4) before and after routine eGFR reporting at an academic, hospital-based primary care (general internal medicine [GIM]) practice. In addition, we examine the care of advanced CKD patients solely managed by their PCPs (hereafter referred to as PCP managed) or co-managed with a nephrologist (hereafter referred to as co-managed) before and after routine eGFR reporting. We hypothesized that following routine eGFR implementation there would be modest improvements in care for PCP managed patients but no improvements in care for co-managed patients. We also hypothesized that co-managed CKD patients would have better kidney-disease specific processes of care compared to PCP managed patients.

## Methods

### Study population and setting

**Figure 1:**
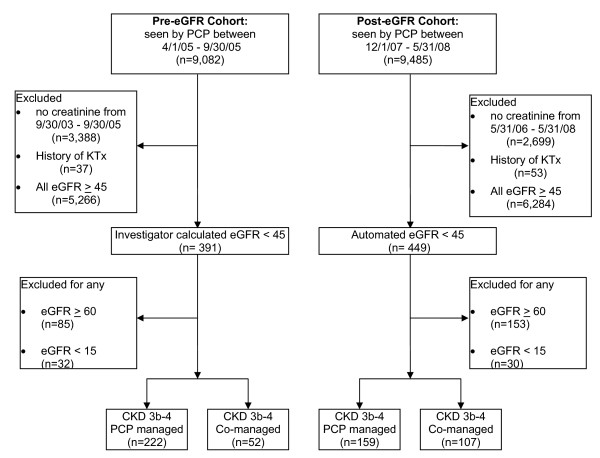
**Patient selection**. eGFR estimated glomerular filtration rate, KTx kidney transplant, CKD chronic kidney disease, PCP primary care physician.

We conducted a retrospective review of University of Pittsburgh Medical Center outpatients cared for at a hospital-based primary care (i.e., GIM) clinic prior to and following routine laboratory implementation of eGFR reporting that began in October 2005. The study inclusion criteria were age ≥ 18 years, the presence of at least one automated or investigator-calculated eGFR value of < 45 ml/min/1.73 m^2^, and an outpatient visit with a university GIM PCP either during the 6-months immediately prior to local laboratory eGFR reporting (pre-eGFR cohort 4/1/05-9/30/05) or during 6-months after local eGFR reporting had become routine (post-eGFR cohort 12/1/07-5/31/08, Figure 1). We chose a more recent 6-month post-eGFR period to allow the measure to gain wider acceptance and to give providers the opportunity to acclimate to its use. Participants were excluded for the following reasons: prior kidney transplant, any form of renal replacement therapy, CKD stage 5 (eGFR < 15 ml/min/1.73 m^2^), or any automated or investigator calculated eGFR ≥ 60 ml/min/1.73 m^2^. The University of Pittsburgh Institutional Review Board approved this study.

### Data collection

#### Creatinine/eGFR ascertainment and reporting

For all eligible participants, all creatinine and eGFR values were abstracted from the electronic medical record (EMR). For the pre-eGFR cohort, all serum creatinine values from 9/30/03 - 9/30/05 were abstracted. For the post-eGFR cohort, all eGFR and serum creatinine values from 5/31/06 - 5/31/08 were abstracted. Non-calibrated, modified Jaffe serum creatinine assays were used by the local university laboratory during both study periods. The 4-variable MDRD study equation [18] was used by either the study investigators or the local laboratory to calculate eGFR values for the pre-eGFR cohort and the post-eGFR cohort, respectively.

For all patients with a laboratory reported eGFR, the following message accompanied their eGFR results, "eGFR < 60 mL/min/1.73 m^2 ^indicates kidney disease. eGFR < 15 mL/min/1.73 m^2 ^indicates kidney failure." No systematic educational activities regarding eGFR reporting were undertaken at the university hospital prior to or following eGFR implementation as of 5/31/2008.

#### Outcomes

The primary outcome was referral to a nephrologist determined by the presence of an EMR order for a nephrology consultation (regardless of whether the patient kept the appointment) or the presence of a nephrology encounter in the EMR during the six month cohort period or within 2 months of the end of each respective cohort period. This 2-month delay allowed time for patients evaluated at the end of the 6-month period to have laboratory tests completed and further orders placed (e.g., specialist referral) as deemed appropriate by the PCP.

Secondary outcomes included CKD documentation, ACEI or ARB use, non-steroidal anti-inflammatory drug (NSAID) use, urinary albumin quantification, and lab monitoring of hemoglobin (Hgb), phosphorus, and parathyroid hormone (PTH) levels. We also intended to assess serum calcium monitoring; however, in early 2006 serum calcium was included in the laboratory's basic metabolic profile confounding any possible pre-eGFR versus post-eGFR comparisons. CKD documentation was determined by the presence of an International Classification of Disease, Ninth Revision (ICD-9) code on the patient's EMR problem list or as a billing diagnosis in a PCP outpatient encounter. These codes included: 249.4(×), 250.4(×), 403.(×), 404.(×), 581.(×), 582.(×), 585.(×), 586, 587, 588.(×), 593.7×, 753.13, 753.14, and 794.4. Use of an ACEI/ARB was determined by the presence of an ACEI/ARB on the patient's EMR medication list at the end of each cohort period. The EMR medication list automatically updates based on electronic prescriptions or medications entered for documentation purposes. Use of an NSAID was determined by the presence of an NSAID on the patient's EMR medication list at the end of each respective cohort period. Urinary albumin quantification was determined by the presence of an EMR order or laboratory value for a random, quantitative spot assessment for albuminuria in the prior 12 months. Lab testing for Hgb, phosphorus, and PTH was determined by the presence of an EMR order or laboratory value for each respective test within the prior 12 months.

#### Covariates

We abstracted the following covariates from the EMR on all eligible patients: age, sex, race (self-reported), insurance status, diagnosis of diabetes, diagnosis of hypertension, and baseline co-management status (i.e., baseline renal referral status). Insurance status was categorized as private, Medicare, medical assistance/Medicaid, or self-pay. Diabetes and hypertension were determined by the presence of the disease on the patient's EMR problem list or as a billing diagnosis in a PCP outpatient encounter in the 12 months preceding the end of each cohort period. Baseline co-management status was determined by the presence of any EMR outpatient encounter with a nephrologist during the 24 months preceding each cohort period. A simple random sample was used to select 10% of patient charts for an EMR chart audit to verify the accuracy of abstracted data.

### Statistical analyses

Differences between the groups in demographic characteristics and clinical variables were assessed using a Student's *t*-test or analysis of variance for continuous variables and a chi-square or Fisher's exact test for categorical variables. Differences in clinical care (nephrology referral, CKD documentation, ACEI/ARB use, etc) were assessed using a chi-square or Fisher's exact test for categorical variables. For the primary outcome (nephrology referral), associations with demographic and clinical variables were assessed in the pre- and post-eGFR cohorts using a chi-square test. Multivariable associations between the dependent variable, co-management status, and demographic and clinical variables were examined in each cohort using a logistic regression model. Model selection was based on variables that were found to have an association (P < 0.2) in univariate analyses. Final model variables were age, sex, race, insurance status, diabetic status, and creatinine. For all analyses, P values < 0.05 were considered significant. Analyses were performed using SAS version 9.1 (Cary, NC).

## Results

### Baseline characteristics

In the pre-eGFR cohort, 391 eligible patients had an eGFR < 45 ml/min/1.73 m^2 ^(Figure 1). Just over 79% of these patients had 2 or more serum creatinine measurements. In the post-eGFR cohort, 449 eligible patients had an eGFR < 45 ml/min/1.73 m^2 ^(Figure 1). Over 94% had 2 or more serum creatinine measurements. After excluding patients with any eGFR < 15 ml/min/1.73 m^2 ^or ≥ 60 ml/min/1.73 m^2^, 274 and 266 patients remained in the pre-eGFR and post-eGFR cohorts, respectively (Figure 1).

Patients in the post-eGFR cohort were more likely to be male, African-American, and have hypertension documented in the EMR than pre-eGFR patients (Table 1). Additionally, post-eGFR patients were more likely to have private insurance and less likely to have Medicare; they also had higher serum creatinines and modestly lower eGFR values (Table 1).

**Table 1:** Baseline characteristics

	Pre-eGFR (n = 274)	Post-eGFR (n = 266)	P-Value*
**Age **(years)	70.5 (13.7)	68.7 (13.4)	0.12

**Female**	70.8% (194)	60.9% (162)	0.02

**African-American**	23.7% (65)	38.3% (102)	0.0002

**Diabetes**	31.8% (87)	36.8% (98)	0.21

**Hypertension**	63.1% (173)	79.7% (212)	< 0.0001

**Insurance Status**			

Private	32.5% (89)	46.6% (124)	0.002

Medicare	56.6% (155)	45.9% (122)	

Medical Assistance	5.5% (15)	5.6% (15)	

Self-pay	5.5% (15)	1.9% (5)	

**eGFR **(ml/min/1.73 m^2^)	36.1 (7.1)**	33.7 (7.9)^‡^	0.0002

**Creatinine **(mg/dl)	1.80 (0.52)**	1.97 (0.72)^‡^	0.002

Continuous variables are presented as means with standard deviations. Categorical variables are expressed as percentages and frequencies.

*Students t-test, chi-square, or Fisher's exact test used as appropriate.

eGFR estimated glomerular filtration rate.

When examining patients stratified by eGFR cohort and referral status (Table 2), PCP managed patients in the pre-eGFR cohort were older, less likely to be African-American, less likely to have hypertension documented in the EMR, less likely to have private insurance and more likely to have Medicare than PCP managed patients in the post-eGFR cohort. PCP managed patients in the pre-eGFR cohort also had modestly lower serum creatinines and higher eGFR values than PCP managed post-eGFR patients.

**Table 2:** Baseline characteristics by eGFR and co-managed status

	Pre-eGFR/ PCP managed (n = 222)	Post-eGFR/ PCP managed (n = 159)	P-Value* (PCP managed, Pre vs. Post)	Pre-eGFR/ Co-managed (n = 52)	Post-eGFR/ Co-managed (n = 107)	P-Value* (Co- managed, Pre vs. Post)
**Age **(years)	72.2 (12.5)**^†^**	69.6 (13.0)	0.05	63.3 (16.1)**^†^**	67.3 (14.0)	0.11

**Female**	72.1% (160)	62.9% (100)	0.06	65.4% (34)	57.9% (62)	0.37

**African-American**	22.5% (50)	36.5% (58)	0.0005	28.9% (15)	41.1% (44)	0.13

**Diabetes**	30.2% (67)	34.0% (54)	0.25	38.5% (20)	41.1% (44)	0.75

**Hypertension**	64.4% (143)	75.5% (120)	0.02	57.7% (30)	86.0% (92)	< 0.0001

**Insurance Status**						

Private	30.6% (68)	45.3% (72)	0.009	40.4% (21)	48.6% (52)	0.67
			
Medicare	59.0% (131)	49.1% (78)		46.2% (24)	41.1% (44)	
			
Medical Assistance	5.0% (11)	4.4% (7)		7.7% (4)	7.5% (8)	
			
Self-pay	5.4% (12)	1.3% (2)		5.8% (3)	2.8% (3)	

**eGFR** (ml/min/1.73 m^2^)	37.2 (6.3)**	35.5 (7.5)^‡^	0.02	31.4 (8.2)**	31.0 (7.6)^‡^	0.78

**Creatinine **(mg/dl)	1.71 (0.42)**	1.83 (0.56)^‡^	0.02	2.18 (0.71)**	2.19 (0.86)^‡^	0.94

Continuous variables are presented as means with standard deviations. Categorical variables are expressed as percentages and frequencies.

*Students t-test, chi-square, or Fisher's exact test used as appropriate.

**^†^**pre-eGFR PCP managed versus pre-eGFR co-managed (P = 0.0004)

**pre-eGFR PCP managed versus pre-eGFR co-managed (P < 0.0001)

‡post-eGFR PCP managed versus post-eGFR co-managed (P < 0.0001)

eGFR estimated glomerular filtration rate, PCP primary care physician.

Co-managed patients in the pre-eGFR cohort were less likely to have hypertension documented in the EMR than co-managed post-eGFR patients (Table 2).

Pre-eGFR PCP managed patients were significantly older than pre-eGFR co-managed patients (Table 2). In addition, PCP managed patients had lower serum creatinines and higher eGFR values than co-managed patients regardless of their eGFR cohort.

### Renal referrals and co-management

**Figure 2:**
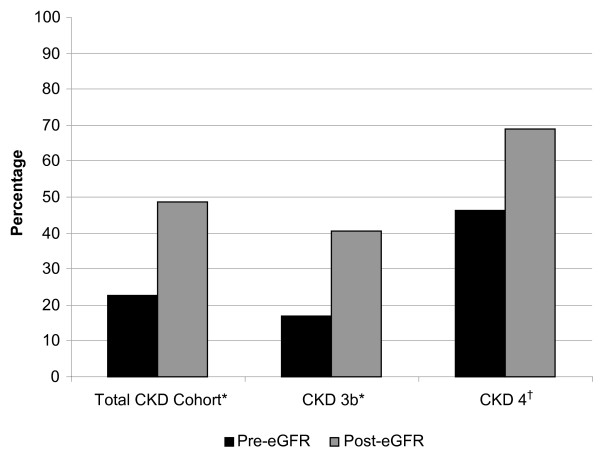
**Co-management of CKD patients by eGFR cohort**. Pre-eGFR cohort: N=274, Post-eGFR cohort: N=266 *P<0.0001, †P=0.01 CKD chronic kidney disease, eGFR estimated glomerular filtration rate

The overall prevalence of CKD 3b-4 co-management increased significantly from the end of the pre-eGFR cohort (22.6%) to the end of the post-eGFR cohort (48.5%, P < 0.0001; Figure 2). Differences in the prevalence of co-managed patients were apparent in both the CKD 3b and CKD 4 subgroups (Figure 2). In addition, a smaller proportion of PCP managed patients were newly referred during the 8 month study/follow-up period in the pre-eGFR cohort versus the post-eGFR cohort; however, the absolute percentage of patients newly referred was modest in both arms (pre-eGFR 4.5% vs. post-eGFR 13.8%, P = 0.001). The percentage of patients newly referred to a nephrologist during the study period was mildly higher when examining the CKD 4 subgroup (pre-eGFR 9.4% [n = 32] vs. post-eGFR 25.8% [n = 31], P = 0.09). Excluding all patients with a single creatinine value did not substantially alter our findings. Examining only patients with 3 or more serum creatinine values did not qualitatively alter our findings although a modest increase in co-management was noted in both the pre-eGFR and post-eGFR groups.

We examined univariate associations with renal referral status at the end of each cohort period. In the pre-eGFR cohort, age < 70 (35% vs. 15%, P < 0.0001), African-American race (34% vs. 19%, P = 0.01), and CKD documentation in the EMR (50% vs. 9%, P < 0.0001) were associated with increased renal referrals. In the post-eGFR cohort, male gender (57% vs. 43%, P = 0.03), diabetic status (57% vs. 44%, P = 0.03), and CKD documentation in the EMR (79% vs. 17%, P < 0.0001) were associated with increased referrals.

After adjusting for serum creatinine, a multivariable analysis of referral status revealed that only age < 70 years was associated with renal referrals in the pre-eGFR cohort while only diabetic status was borderline associated with referrals in the post-eGFR cohort (Table 3). CKD documentation was excluded from these models due to concern that nephrologists may have been responsible for documenting CKD in the EMR problem list after a patient was referred and evaluated.

**Table 3:** Multivariable associations with co-managed status after adjusting for serum creatinine

	Pre-eGFR cohort*	Post-eGFR cohort^†^
	Adjusted OR (95%CI)	Adjusted OR (95%CI)

Age (< 70 vs. ≥ 70 years)	**2.74 (1.37-5.49)**	1.49 (0.84-2.66)

Sex (female vs. male)	1.48 (0.65-3.36)	0.99 (0.54-1.84)

Race (AA vs. non-AA)	1.05 (0.49-2.27)	0.85 (0.47-1.55)

Insurance Status (private vs. non-private**)	1.81 (0.88-3.76)	1.16 (0.65-2.07)

Diabetes (diabetic vs. non-diabetic)	1.61 (0.81-3.19)	1.77 (1.00-3.14)

For each variable, the referent is the latter value listed within parentheses.

*Pre-eGFR cohort: N = 274;

**^†^**Post-eGFR cohort: N = 266;

**Non-private insurance included Medicare, medical assistance/Medicaid, or self-pay.

eGFR estimated glomerular filtration rate, AA: African-American.

### Trends in CKD care

Overall, ACEI/ARB use, urinary albumin quantification, absence of both ACEI/ARB and urinary albumin quantification, and Hgb monitoring did not improve following eGFR reporting (58.4% vs. 58.6%, P = 0.9; 24.5% vs. 29.3%, P = 0.2; 37.6% vs. 32.7%, P = 0.2; 80.3% vs. 86.1%, P = 0.07, respectively). However, NSAID avoidance and phosphorus and PTH monitoring improved following routine eGFR reporting (81.8% vs. 90.6%, P = 0.003; 32.5% vs. 51.5%, P < 0.0001; 12.4% vs. 36.1%, P < 0.0001, respectively). In sensitivity analyses examining patients with at least 2 or at least 3 serum creatinine values, findings did not substantially differ.

**Figure 3:**
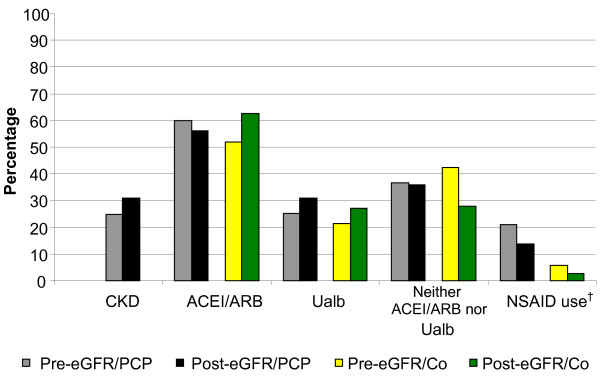
**Quality of care delivered to CKD 3b-4 patients**. Pre-eGFR cohort: n=274, Post-eGFR cohort: n=266. † Pre-eGFR/PCP vs. Post-eGFR/PCP, P=0.07; Pre-eGFR/PCP vs. Pre-eGFR/Co, P=0.01; PosteGFR/  PCP vs. Post-eGFR/Co, P=0.003. CKD chronic kidney disease documentation, ACEI/ARB angiotensin converting enzyme  inhibitor or angiotensin receptor blocker use, NSAID non-steroidal anti-inflammatory drug, Ualb  quantitative urinary albumin testing, eGFR estimated glomerular filtration rate, PCP primary  care physician managed, Co co-managed.

**Figure 4:**
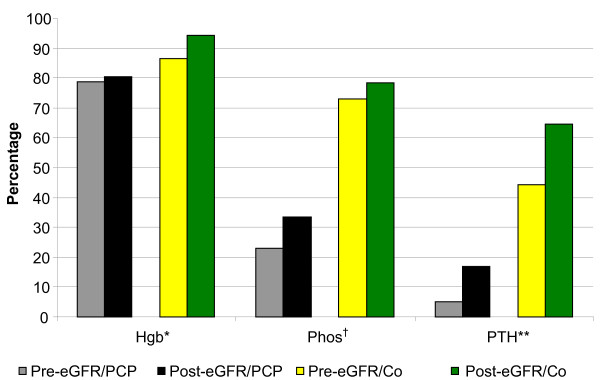
**Laboratory processes of care for CKD 3b-4 patients**. Pre-eGFR cohort: n=274, Post-eGFR cohort: n=266. * Post-eGFR/PCP vs. Post-eGFR/Co, P=0.001. †Pre-eGFR/PCP vs. Post-eGFR/PCP, P=0.03; Pre-eGFR/PCP vs. Pre-eGFR/Co, P<0.0001; PosteGFR/  PCP vs. Post-eGFR/Co, P<0.0001. **Pre-eGFR/PCP vs. Post-eGFR/PCP, P=0.0001; Pre-eGFR/Co vs. Post-eGFR/Co, P=0.02; PreeGFR/  PCP vs. Pre-eGFR/Co, P<0.0001; Post-eGFR/PCP vs. Post-eGFR/Co, P<0.0001.  Hgb hemoglobin, Phos phosphorus, PTH parathyroid hormone, eGFR estimated glomerular  filtration rate, PCP primary care physician managed, Co co-managed.

When examining PCP managed patients, there was no difference in CKD documentation, ACEI/ARB use, urinary albumin quantification, absence of ACEI/ARB and urinary albumin quantification, or Hgb monitoring following the use of eGFR reporting (Figure 3 and Figure 4). However, there was a borderline significant decrease in NSAID use and significant improvements in phosphorus and PTH monitoring following eGFR implementation (Figure 3 and Figure 4).

When examining co-managed patients, only PTH monitoring improved following eGFR reporting (Figure 3 and Figure 4). However, ACEI/ARB use in co-managed CKD 4 patients rose from 36.4% (n = 22) pre-eGFR to 60.5% (n = 43) post-eGFR, although this was not statistically significant (P = 0.07).

When comparing PCP managed and co-managed patients, NSAID use was higher and phosphorus and PTH monitoring were lower in PCP managed patients regardless of eGFR reporting (Figure 3 and Figure 4). In addition, in the post-eGFR cohort, PCP managed patients were less likely to obtain Hgb testing than co-managed patients (Figure 4).

## Discussion

We observed a substantial increase in CKD patient co-management following the use of routine eGFR reporting at an academic, hospital-based GIM clinic. At the conclusion of the respective study periods, nearly 50% of CKD 3b-4 patients were co-managed in the post-eGFR cohort compared to less than 25% of the pre-eGFR cohort. Significant increases in co-management were apparent in both CKD 3b and CKD 4 patients. Following eGFR implementation, we also noted significant improvements in NSAID avoidance and mineral and bone disease related lab testing. However, ACEI/ARB use and urinary albumin quantification were not improved. While CKD care delivery to both PCP managed and co-managed patients remained suboptimal after eGFR reporting, co-managed patients were less likely to be on NSAIDs and more likely to have lab testing for complications of CKD.

Our observed increase in CKD patient co-management is similar to previous reports documenting increased nephrology referrals following implementation of routine eGFR reporting [8,26-28]. At our institution, moderate to advanced CKD patients previously had their kidney disease managed predominantly by their PCPs. Now, nearly half of all CKD 3b-4 patients are being co-managed. We noted both an increase in the prevalent proportion of CKD 3b-4 patients who were co-managed at the outset of each cohort as well as a significant increase in the proportion of patients who were newly referred during the study period. Together, these increases in renal referrals represent a substantial shift in CKD care by PCPs. Such shifts in PCP practice patterns may pose a logistical challenge to nephrology groups that are not equipped to deal with an influx of pre-dialysis CKD patients. Other centers have successfully adopted education and referral guideline programs to address this problem when present [8,36]. However, in localities where the increase in CKD patient referrals does not overwhelm capacity, this shift provides nephrologists with an opportunity to enhance the care of many CKD patients at an earlier stage of disease when treatment is more likely to prevent or delay cardiovascular disease and kidney failure [16,37-39].

Increased CKD co-management should lead to a greater emphasis on ensuring that treatment delivered by PCPs and nephrologists represents optimal care to reduce CKD morbidity and mortality. In addition to improved communication between PCPs and nephrologists, important goals in the management of CKD patients include blood pressure control, proteinuria suppression, ACEI/ARB therapy, cardiovascular disease risk factor modification, nephrotoxin avoidance, and treatment of complications from CKD (e.g., anemia) [7,40-42]. Although we did not examine clinical outcomes in this study, it is notable that ACEI/ARB use and lack of both albuminuria screening and ACEI/ARB therapy were unaffected by eGFR reporting. While a trend towards improved ACEI/ARB use was seen in co-managed CKD stage 4 patients, we suspect this was due to recent literature documenting the safety and efficacy of ACEI treatment in the setting of advanced CKD that was not available during the pre-eGFR cohort [43].

Our findings are similar to previous studies reporting minimal or no improvement in ACEI/ARB use following eGFR implementation [28,29]. In addition, approximately 1/3 of our CKD cohort received neither an albuminuria screen nor ACEI/ARB therapy. Hence, providers did not know whether a significant number of their CKD patients possessed characteristics making them likely to benefit from ACEI/ARB therapy nor were they presumptively treating such patients. Although contraindications to ACEI/ARB therapy (e.g., angioedema, severe hyperkalemia) may have precluded their use, it should be noted that published rates of hyperkalemia in a recent non-dialysis dependent CKD 3-5 cohort were approximately 5-12% [44] and the approximate rate of hyperkalemia (K > 5.5) for outpatients with CKD 3b-5 at our medical center is 17.1% in the previous 12 months (unpublished data). Hence, contraindications to ACEI/ARB are unlikely to explain these shortcomings in care fully.

While differences in CKD care between PCP managed and co-managed patients were apparent in the rates of NSAID avoidance and lab monitoring for potential complications of CKD, our findings generally confirm studies documenting sub-optimal CKD care among PCPs and nephrologists prior to eGFR reporting [10,33-35,45]. These results suggest that even with eGFR reporting, there remains substantial room for improvement in CKD care. Despite the marked increase in co-management, the majority of patients who were PCP managed at the outset of each cohort were not referred over the subsequent 8-months. This is similar to findings by Richards et al [8] who noted that despite the marked increase in renal referrals following eGFR implementation, only 33% of non-referred CKD 4-5 patients were referred in the following 12 months. This implies that although referrals have generally increased following eGFR reporting, a subset of patients may still be referred late. In our multivariable adjusted model of co-management status following eGFR reporting, only creatinine and diabetic status were associated with co-management. Given the documented increase in morbidity and mortality associated with late referrals [46], future studies should further examine patient characteristics that are associated with late referrals despite eGFR reporting.

Our findings should be interpreted in light of several limitations. First, we only examined patients with a documented creatinine value; however, some patients with CKD 3b-4 are unscreened and would be excluded from our analysis. Hence, the true rate of co-management and appropriate process of care outcomes is likely to be lower than reported here. Second, we required one eGFR < 45 ml/min/1.73 m^2 ^for inclusion in this study. However, the current National Kidney Foundation CKD definition requires a reduction in the GFR to < 60 ml/min/1.73 m^2 ^for at least 3 months. This potentially resulted in misclassification. However, the presence of two or more calculated eGFR values in nearly 80% and 95% of patients in the pre-eGFR and post-eGFR cohorts respectively and the exclusion of all patients with a recent eGFR ≥ 60 ml/min/1.73 m^2 ^should have minimized this bias. Further, exclusion of patients with only 1 or 2 creatinine/eGFR measures did not significantly alter our findings. Third, serum creatinine assays were not standardized during the study period. Fourth, there were additional important process of care outcomes that we did not assess including appropriate lab monitoring following the initiation of ACEI/ARB therapy. Fifth, improvements in care could be due to secular trends rather than eGFR implementation. For example, the recent interest in mineral and bone disease including vitamin D deficiency could explain the increased monitoring of phosphorus and PTH. In addition, there are other critical factors that may contribute to differences in the care delivered to CKD patients that were not controlled for in this study (e.g., patient comorbidities, underlying etiology of CKD). Finally, we studied a relatively small sample.

## Conclusions

Following the implementation of routine eGFR reporting at an academic, outpatient primary care clinic, there has been a marked increase in the co-management of CKD 3b-4 patients and modest to moderate improvement in CKD processes of care including NSAID avoidance and mineral and bone disease related lab testing. However, pre-dialysis CKD care remains suboptimal. While the best approach to improve clinical care for CKD patients is unproven, multiple interventions will likely be required to affect physician behavior [47]. Simple educational didactics, printed materials, and guideline development alone have generally failed to change practice patterns in a variety of medical settings [47]. Alternative approaches including combinations of audit and feedback, educational outreach, and clinical decision support systems have garnered increasing attention as potentially effective tools to improve physician performance [48-53].

This study identifies not only PCP care as suboptimal, but also reinforces prior documented shortcomings in nephrology care [10,33-35,45]. Such deficiencies despite the presence of CKD guidelines and eGFR reporting, especially in an academic setting where patient visits are generally longer and providers less hurried, highlight the need to employ a conceptual model of suboptimal CKD care delivery by providers to direct the evaluation of further interventions that may improve care delivery. Such a model should identify the numerous hurdles including perceptual and interpretive errors that may have been addressed by eGFR reporting. It should also identify additional barriers including gaps in knowledge and cognitive burdens not addressed by eGFR reporting but that will need to be overcome to incrementally enhance CKD care. Further studies are needed to explore the role of systematic interventions in addressing these barriers and optimizing the care of pre-dialysis CKD patients.

## Competing interests

The authors declare that they have no competing interests.

## Authors' contributions

KA, GF, JJ, CM, and MU conceptualized the study. KA, GF, CM, and MU obtained funding. KA, GF, CG, CM, and MU acquired the data. KA, CG, CM, and MU analyzed the data. KA, GF, JJ, CG, CM, and MU contributed to interpretation and manuscript preparation. All authors read and approved the final manuscript.

## Pre-publication history

The pre-publication history for this paper can be accessed here:

http://www.biomedcentral.com/1471-2369/12/12/prepub
